# Targeted Inactivation of *Rin3* Increases Trabecular Bone Mass by Reducing Bone Resorption and Favouring Bone Formation

**DOI:** 10.1007/s00223-021-00827-2

**Published:** 2021-03-16

**Authors:** Mahéva Vallet, Antonia Sophocleous, Anna E. Törnqvist, Asim Azfer, Rob van’t Hof, Omar ME Albagha, Stuart H. Ralston

**Affiliations:** 1 Institute of Genetics and Molecular Medicine, University of Edinburgh, Edinburgh, UK; 2 Department of Life Sciences, School of Sciences, European University Cyprus, Engomi, Cyprus; 3 Centre for Bone and Arthritis Research, Institute of Medicine, University of Gothenburg, Gothenburg, Sweden; 4 Institute of Ageing and Chronic Disease, University of Liverpool, Liverpool, England; 5 College of Health and Life, Hamad Bin Khalifa University, Doha, Qatar

**Keywords:** Paget’s disease, Osteoclast, Genetic, RIN3

## Abstract

Common genetic variants at the *RIN3* locus on chromosome 14q32 predispose to Paget’s disease of bone (PDB) but the mechanisms by which they do so are unknown. Here, we analysed the skeletal phenotype of female mice with targeted inactivation of the mouse *Rin3* gene (*Rin3*^*−/−*^) as compared with wild-type littermates. The *Rin3*^*−*/*−*^ mice had higher trabecular bone volume (BV/TV%) compared with wild type. Mean ± standard deviation values at the distal femur at 8 weeks were 9.0 ± 2.5 vs. 7.0 ± 1.5 (*p* = 0.002) and at 52 weeks were 15.8 ± 9.5 vs. 8.5 ± 4.2 (*p* = 0.002). No differences were observed in femoral cortical bone parameters with the exception of marrow diameter which was significantly smaller in 52-week-old *Rin3*^*−*/*−*^ mice compared to wild type: (0.43 mm ± 0.1 vs. 0.57 mm ± 0.2 (*p* = 0.001). Bone histomorphometry showed a lower osteoclast surface / bone surface (Oc.S/BS%) at 8 weeks in *Rin3*^*−*/*−*^ mice compared to wild type (24.1 ± 4.7 vs. 29.7 ± 6.6; *p* = 0.025) but there were no significant differences in markers of bone formation at this time. At 52 weeks, Oc.S/BS did not differ between genotypes but single labelled perimeter (SL.Pm/B.Pm (%)) was significantly higher in *Rin3*^*−*/*−*^ mice (24.4 ± 6.4 vs. 16.5 ± 3.8, *p* = 0.003). We conclude that *Rin3* negatively regulates trabecular bone mass in mice by inhibiting osteoclastic bone resorption and favouring bone formation. Our observations also suggest that the variants that predispose to PDB in humans probably do so by causing a gain-in-function of *RIN3*.

**Supplementary Information:**

The online version of this article (10.1007/s00223-021-00827-2) contains supplementary material, which is available to authorised users.

## Introduction

Paget’s disease of bone (PDB) is characterised by increased bone remodelling at one or more skeletal sites. It is thought that the primary pathogenic event in PDB is increased osteoclast activity, but this is accompanied by increased and disorganised bone formation. Genetic factors play a key role in the pathogenesis of PDB, but environmental triggers also play a role as reflected by a reduction in the prevalence and severity of PDB in many countries over recent decades [1]. Genome-wide association studies (GWAS) have so far identified six loci that predispose to PDB at a genome-wide significant level [2, 3], and additional predisposing genes have been identified by linkage analysis and genome sequencing in families [4–6]. A locus on chromosome 14q32 was found to predispose to PDB by an extended GWAS [2]. The strongest association was observed with rs10498635 which is situated close to the *RIN3* gene, encoding the Ras and Rab interactor protein 3 [7]. The rs10498635 variant lies outside the gene but is in strong linkage disequilibrium with an arginine to cysteine amino acid change at position 279 (R279C) of the protein [2]. Subsequent mutation screening of *RIN3* identified several other protein coding variants that were associated with PDB which cluster in a proline-rich domain of the protein [8]. In another study of Belgian PDB patients and controls, de Ridder and colleagues [9] confirmed evidence of association between variants at the *RIN3* locus and PDB and identified several rare potentially deleterious coding variants that were associated with the disease. While the role of Ras and Rab interactor *3* in bone metabolism remains unclear, it is a guanine exchange factor for small GTPases which are known play a role in vesicular trafficking and other processes relevant to osteoclastic bone resorption [10, 11]. With this background, the aim of the present study was to gain an insight into the role of *RIN3* in skeletal homeostasis by conducting skeletal phenotyping of mice with targeted inactivation of the *Rin3* gene.

## Materials and Methods

### Animals

Mice with targeted inactivation of *Rin3* (*Rin3*^*−*/*−*^), generated on a C57BL/6J background, were obtained from the Mutant Mouse Resource and Research Center (MMRC) catalogue number 049460-UCD, through the Jackson Laboratory. Deletion of *Rin3* had been obtained by transfecting embryonic stem cells with the targeting vector containing a Velocigene cassette (ZEN-Ub1). The targeting procedure generated mice with a 29.89Kb deletion at the *Rin3* locus encompassing part of exon 1, all of exon 2 and most of exon 3 of *Rin3*. The *Rin3*^*−*/*−*^ mice were crossed with 129P2OlaHsd mice obtained from Harlan UK to generate *Rin3*^*+*/*−*^ heterozygotes on a mixed C57BL/6J and 129P2OlaHsd genetic background. All experiments were performed on female *Rin3*^*−*/*−*^ and wild-type littermates generated from mating of *Rin3*^*+/-*^ heterozygotes. We confirmed that mRNA for *Rin3* was undetectable in lung tissue from *Rin3*^*−*/*−*^ mice using qPCR, confirming that the targeted inactivation of *Rin3* was successful (data not shown). The mice were housed in a designated animal facility in pathogen-free rooms maintained at constant temperature, with 12-h light/12-h dark cycles. They had free access to water and pelleted standard commercial diet (SDS, Special Diets Service). Mice were sacrificed by cervical dislocation or decapitation according to Schedule 1 of the Animals (Scientific Procedures) Act. The sample size for the in vivo experiment was chosen to provide at least 80% power to detect a 1.3 standard deviation difference in BV/TV between *Rin3*^*−*/*−*^ and wild-type (WT) mice.

### Analysis of *Rin3* Gene Expression

In order to assess the efficiency of the targeted inactivation procedure, RNA was extracted from homogenised lung tissue of 7.5-week-old mice using TRizol® reagent (Invitrogen). Complementary DNA (cDNA) was transcribed by reverse transcription-polymerase chain reaction (RT-PCR) using the SuperScript III Reverse Transcriptase kit (Invitrogen). The expression *Rin3* was assessed by quantitative PCR with the Roche universal library probe system (Roche). A FAM-labelled probe (#13, AGGCAGAG) was used with the following manually designed primers from the Ensembl Genome Browser: forward 5′-GCCGGTCCTATTCCAGATG-3′; Reverse 5′-AAGAACTGAGCCTTCCAGGTA-3′. The reaction was performed using the SensiFAST Probe kit (Bioline) on a Chromo 4TM thermocycler (M J Research). The levels of *Rin3* were normalised against eukaryotic 18S ribosomal RNA, which was detected using a VIC-labelled probe-primer (Applied Biosystems).

### Transcriptome Analysis by RNA Sequencing

We evaluated global gene expression in bone marrow derived osteoclast cultures and primary calvarial osteoblast cultures derived from *Rin3*^*−*/*−*^ and wild-type mice. The cultures were prepared from three mice of each genotype for each cell type and total RNA was extracted using TRizol® reagent (Invitrogen). The TruSeq Stranded Total RNA library Gold prep kit (Illumina) was used for preparation of the cDNA library and sequencing was performed using an Illumina HiSeq 4000 75PE to yield 290 Million + 290 Million reads per lane in 2 lanes. Full details of the methods used to process the data and to analyse gene expression are provided in the supplementary information.

### MicroCT Analysis

Bone density and structure were analysed by micro computed tomography (microCT) using a Skyscan 1172 instrument (Bruker, Belgium). Prior to analysis, the bone samples were dissected free of soft tissue and fixed in 4% (v/v) paraformaldehyde in PBS overnight. The samples were then washed twice in PBS and stored at 4 °C in 70% (v/v) ethanol until scanning. Analysis of trabecular bone was performed by scanning the distal femoral and proximal tibial metaphysis and the 6th lumbar vertebra. At the distal femoral metaphysis, 450 slices were analysed in a region of interest 750 µm from the growth plate. At the proximal tibial metaphysis, 200 slices were analysed in a region of interest 500 µm from the growth plate. At lumbar vertebra, 200 slices were analysed 250 µm distally the transverse process. Analysis of cortical bone was performed on the femoral diaphysis only, where 200 slices were analysed 5 mm from the growth plate. The samples were scanned using an X-Ray source set at 60 kV and 167 µA, a 0.5 mm aluminium filter and a resolution of 5 µm. The cross-section images were reconstructed using the NRecon system (Bruker, Belgium). The transaxial view of this model was then analysed with a free hand tracing tool at the region of interest, using the CTAn system (Bruker, Belgium). Both hind legs were used for the trabecular and cortical analyses.

### Bone Histomorphometry

Bone samples were processed and stained for histology using standard methods as previously described [12]. Briefly, animals received intraperitoneal injections of calcein 6 days and 2 days before culling. The skin was removed, the bone samples were fixed for 24 h in 4% paraformaldehyde and stored at 4 °C in 70% ethanol until use. The samples were embedded in methyl methacrylate and 5 µm sections were cut using a diamond knife. For analysis of bone resorption parameters, sections were stained for tartrate resistant acid phosphatase (TRAcP) to visualise osteoclasts and counterstained with aniline blue. For analysis of calcein double labelling, sections were counterstained with calcein blue. The bone sections were analysed using a Zeiss LSM800 (Carl Zeiss Ltd., UK) fluorescence microscope. Histomorphometric measurements were performed using an open source software programme [13] by an observer blinded to genotype.

### Biochemical Markers

We measured serum levels of the procollagen I N-terminal propeptide (PINP) and the type I Collagen Cross-Linked C-Telopeptide (CTX-I) by enzyme-linked immunosorbent assay using kits purchased from Immunodiagnostic Systems (IDS) (Boldon UK). Catalogue numbers were AC33F1 (PINP) and AC-06F (CTX-I) and they assays were run according to the manufacturer’s instructions. For both assays, values were recorded based on mean of the duplicate samples and expressed in ng/ml by calibrating against a standard curve.

### Osteoclast Cultures

Osteoclasts were generated from bone marrow cells flushed from the long bones of female mice aged between 9 and 14 weeks. The marrow samples were cultured for 2 days in alpha-MEM supplemented with 1% L-Glutamine, 100 U/ml penicillin, 100 μg/ml streptomycin, 10% foetal calf serum (Hyclone) and 100 ng/ml M-CSF (R&D systems Abingdon UK). Non-adherent cells were removed and the adherent macrophages were plated at 1 × 10^4^ cells in 96-well plates in the presence of 25 ng/ml M-CSF and 75 ng/ml RANKL (R&D Systems) for another 3 days. The medium was changed again in the same conditions and the cells were kept for another 24 h in the incubator. At the end of the culture period, the cells were fixed in 4% paraformaldehyde and stained for TRAcP according to standard techniques. Cells with at least three nuclei that stained positive for TRAcP were counted as osteoclasts. Osteoclasts that contained ≥10 nuclei were considered to be large osteoclasts.

We also studied osteoclast survival in the same cultures. For this assay, RANKL was added at a reduced concentration of 25 ng/ml for the last 4 days of the culture and then the medium was replaced with standard culture medium without RANKL. The cultures continued for up to 72 h from when the cells were fixed, then stained for TRAcP and counted as described above.

### Osteoblast Cultures

Primary osteoblasts were isolated from the calvarial bones of 2-day-old mice using sequential collagenase digestion as previously described [14]. The cells were cultured in 75 cm^2^ flasks in standard alpha-MEM supplemented with 10% foetal bovine serum and left to adhere overnight. To measure the activity of alkaline phosphatase (ALP), the osteoblasts were passaged using trypsin and seeded into 96-well plates at a density of 8 × 10^3^ cells per well. The cells were cultured for 7 days and the experiment was terminated by the addition of lysis buffer containing 1M diethanolamine, 1 mM MgCl_2_, and 0.05% Triton X100. The ALP activity was assessed on cell lysates using a colorimetric assay based on conversion of p-nitrophenyl phosphate to p-nitrophenol using a plate-reader set at an absorbance of 405 nm. Activity of ALP was corrected for cell number following staining with 10% Alamar blue reagent (v/v) as described [15]. We also assessed formation of mineralised bone nodules using calvarial osteoblast cultures. For this assay, the osteoblasts were plated in 12-well plates at a density of 1 × 10^5^ cells per well and cultured for 21 days in osteogenic media as described [15]. At the end of the culture period, cell viability was assessed using staining with 10% Alamar blue reagent (v/v). The cells were then fixed in 70% ethanol, washed in PBS, briefly stained with alizarin red and left to dry overnight. The nodules were quantified by destaining the cultures in a 10% (w/v) cetylpyridinium chloride solution prepared in 10mM sodium phosphate (pH 7.0) and measuring absorbance of the extracted stain by spectrophotometry as previously described [16]. The absorbance was measured at 562 nm and compared to a standard curve. The values were corrected for cell number as assessed by alamar blue staining. All colorimetric and fluorescent measures were done using the BioTek Synergy HT plate reader (BioTek, UK).

### Statistical Analysis

Differences between genotype groups for continuous variables were assessed by *t* test for independent samples in individual experiments. For the cell culture experiments a general linear model analysis of variance was used to study the effect of time and genotype in which all the replicates within individual experiments were combined. For this analysis, the variable of interest (osteoclast number, alizarin red staining, alkaline phosphatase) was selected as the dependent variable and (where relevant) time, experiment and genotype entered as the explanatory variables. Statistical analysis was performed using SPSS version 22 or Minitab version 18. A *p* value of less than 0.05 was considered to be statistically significant.

## Results

### General Appearance and Body Weight

The *Rin3*^*−*/*−*^ mice were of similar appearance to wild-type littermates and had a similar body weight at 8 weeks of age (*Rin3*^*−*/*−*^ vs. WT (mean ± SD) = 20.8 ± 2.4 vs. 21.3 ± 1.5 g, *p* = 0.59) and 52 weeks of age (34.1 ± 4.6 vs. 33.4 ± 1.5 g, *p* = 0.60).

### MicroCT Analysis

**Fig. 1:**
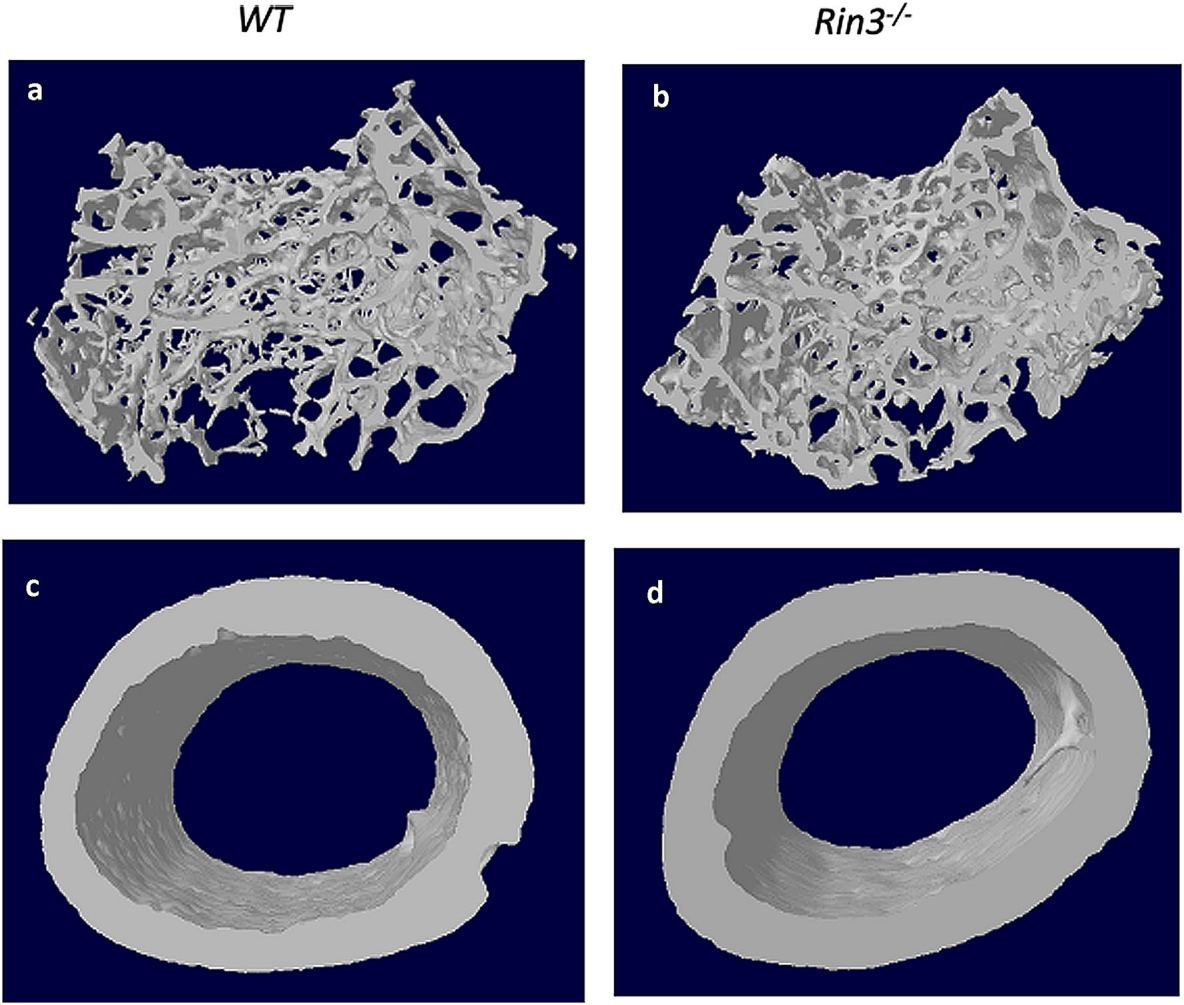
Representative microCT images from *Rin3*^*−*/*−*^ and wild-type mice. Panel **a** trabecular bone in distal femoral metaphysis from wild-type mice (WT); Panel **b** trabecular bone from distal femoral metaphysis *Rin3*^*−*/*−*^ mice; Panel **c** cortical bone from femoral diaphysis in wild-type mice; Panel d: cortical bone from femoral diaphysis in *Rin3*^*−*/*−*^ mice. The microCT images are from 8-week-old mice

The results of microCT analysis of trabecular bone at the femoral metaphysis in 8-week and 52-week-old mice are shown in Table 1. Trabecular bone volume (BV/TV) and trabecular number were significantly higher at the femoral metaphysis of *Rin3*^*−*/*−*^ mice at 8 weeks of age compared with wild-type littermates. Trabecular thickness did not differ between genotypes, but trabecular separation and trabecular patterning factor were both lower in the *Rin3*^*−*/*−*^ mice as compared with wild type. At 52 weeks, the difference between genotypes was even more pronounced with substantially higher BV/TV in the *Rin3*^*−*/*−*^ mice compared to the wild-type littermates. Similar findings were observed at both 8 weeks and 52 weeks at the proximal tibial metaphysis (supplementary information Table S1). Analysis of cortical bone at the femoral diaphysis showed that there was no significant difference in cortical bone mass or structure between *Rin3*^*−*/*−*^ mice and wild-type littermates, except at 52 weeks where the marrow diameter was significantly smaller in *Rin3*^*−*/*−*^ mice compared with wild-type littermates (Table 2). Representative microCT images from the distal femoral metaphysis and the femoral diaphysis in *Rin3*^*−*/*−*^ mice and wild-type littermates at 8 weeks are shown in Fig. 1. In contrast to findings in the limbs, there were no differences in trabecular bone volume or other parameters at the lumbar vertebra 6 in both age groups of *Rin3*^*−*/*−*^ mice compared with wild type (supplementary information Table S2).

**Table 1:** MicroCT analysis of trabecular bone from the distal femoral metaphysis in *Rin3*^*−*/*−*^ and wild-type mice

	8 weeks			52 weeks		
	Wild type (*n* = 11)	*Rin3*^*−*/*−*^ (*n* = 14)	*p* value	Wild type (*n* = 11)	*Rin3*^*−*/*−*^ (*n* = 10)	*p* value
BV/TV (%)	7.0 ± 1.5	9.0 ± 2.5	0.002	8.5± 4.2	15.8 ± 9.5	0.002
Tb.Th (µm)	37.8 ± 1.9	37.2 ± 2.7	0.405	44.2 ± 6.8	47.6 ± 7.1	0.117
Tb.Sp (µm)	261.4 ± 27.7	234.7 ± 26.8	0.001	346.9 ± 93.8	262.0 ± 97.8	0.007
Tb.N (1/mm)	1.85 ± 0.4	2.38 ± 0.5	< 0.001	1.89 ± 0.96	3.22 ± 1.80	0.006
Tb.Pf (1/µm)	0.033 ± 0.00	0.029 ± 0.00	0.002	0.018 ± 0.01	0.014 ± 0.01	0.036

*BV/TV* bone volume/tissue volume, *Tb.Th* trabecular thickness, *Tb.Sp* trabecular separation, *Tb.N* trabecular number, *Tb.Pf* trabecular pattern factor. The values shown are the mean ± SD values. The *p* values refer to differences between genotype groups

**Table 2:** MicroCT analysis of cortical bone from the femoral diaphysis in *Rin3*^*−*/*−*^ and wild-type mice

	8 weeks			52 weeks		
	Wild type (*n* = 11)	*Rin3*^*−*/*−*^ (*n* = 14)	*p* value	Wild type (*n* = 11)	*Rin3*^*−*/*−*^ (*n* = 10)	*p* value
Ct.Dm (mm)	1.56 ± 0.06	1.55 ± 0.08	0.709	1.80 ± 0.10	1.81 ± 0.10	0.787
BV (µm^3^ × 10^8^)	7.52 ± 0.55	7.53 ± 0.82	0.959	15.4 ± 2.50	16.4 ± 2.20	0.185
Ct.Th (µm)	179.5 ± 10.2	180.8 ± 14.3	0.714	307.4 ± 47.4	319.4 ± 33.1	0.344
Ma.Dm (mm)	0.84 ± 0.05	0.82 ± 0.07	0.340	0.57 ± 0.20	0.43 ± 0.10	0.001

*Ct.Dm* cortical diameter, *BV* bone volume, *Ct.Th* cortical thickness, *Ma.Dm* marrow diameter. The values shown are the mean ± SD values from both limbs combined. The *p* values refer to the difference between genotype groups

### Bone Histomorphometry

**Fig. 2:**
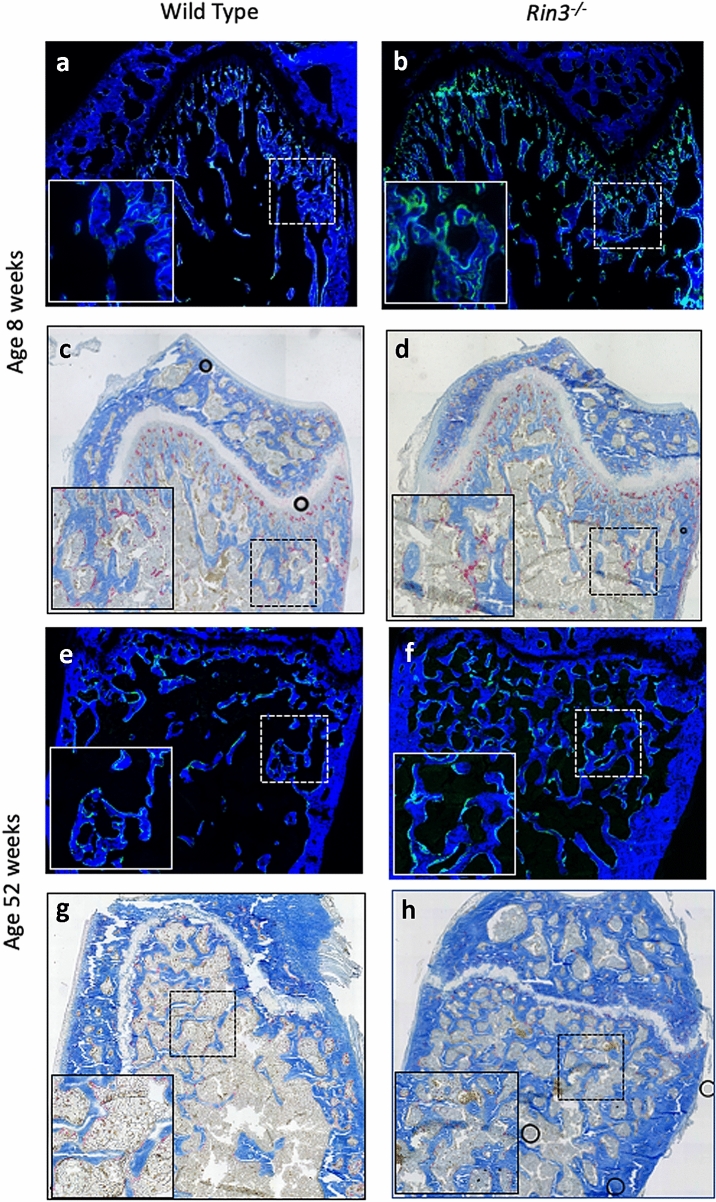
Representative bone histology from *Rin3*^*−*/*−*^ and wild-type mice at aged 8 weeks panels **a**–**d** and 52 weeks (Panels **e**–**h**). Calcein labelling (light green) in panels **a**, **b**, **e** and **f** has been visualised under polarised light. Panels **c**, **d**, **g** and **h** have been stained with aniline blue and counterstained for tartrate resistant acid phosphatase (red). Higher magnification images of the sections are shown in the bottom left of each panel and the area from which the images are taken are indicated by squares with interrupted lines. The black circles in sections **c**, **d** and **h** are air bubbles in the fixative

The results of bone histomorphometry are shown in Table 3. Osteoclast surface expressed as a percentage of bone surface (Os.S/BS) was significantly reduced in 8-week-old *Rin3*^*−*/*−*^ mice compared with wild-type littermates. A similar trend was observed for the number of osteoclasts per unit of bone surface (N.Oc/BS) and for the number of osteoclasts per unit of bone volume (N.Oc/BV), but the differences were not significant for these variables. At 52 weeks, Oc.S/BS, N.Oc/BS and N.Oc/BV were numerically lower in *Rin3*^*−*/*−*^ mice compared wild-type littermates but there was considerable variation and the differences between genotypes was not significant. Mineral apposition rate (MAR) did not significantly differ between genotypes at either 8 weeks or 52 weeks. The perimeter of bone surfaces occupied by single labels (sL.Pm/B.Pm) was significantly higher in *Rin3*^*−*/*−*^ mice at 52 weeks. Representative photomicrographs of trabecular bone from 8-week-old and 52-week-old mice are shown in Fig. 2 which illustrates the difference between genotypes in trabecular bone volume throughout life, the relative reduction in osteoclast surfaces in *Rin3*^*−*/*−*^ mice compared to wild type at 8 weeks of age and the relative increase in single labelled surfaces compared to wild type in *Rin3*^*−*/*−*^ mice at 52 weeks of age.

**Table 3:** Bone histomorphometry in *Rin3*^*−*/*−*^ and wild-type mice

	8 weeks			52 weeks		
	Wild type (*n* = 10)	*Rin3*^*−*/*−*^ (*n* = 13)	*p* value	Wild type (*n* = 12)	*Rin3*^*−*/*−*^ (*n* = 11)	*p* value
Oc.S/BS (%)	29.7 ± 6.6	24.1 ± 4.7	0.025	18.2 ± 5.4	16.2 ± 5.8	0.396
N.Oc/BS (mm^−1^)	12.7 ± 2.7	11.4 ± 2.0	0.221	9.4 ± 2.7	8.4 ± 2.9	0.409
N.Oc/BV (mm^−2^)	845 ± 251	685 ± 172	0.085	432 ± 141	374 ± 185	0.409
MS/BS (%)	38.2 ± 2.7	39.8 ± 5.6	0.436	44.0 ± 4.5	46.9 ± 4.2	0.129
sL.Pm/B.Pm (%)	23.6 ± 4.7	26.9 ± 6.4	0.223	16.5 ± 3.8	24.4 ± 6.4	0.003
dL.Pm/B.Pm (%)	26.3 ± 4.4	26.4 ± 8.3	0.976	35.6 ± 5.5	34.7 ± 5.9	0.719
MAR (µm/day)	1.25 ± 0.20	1.12 ± 0.08	0.091	1.83 ± 0.25	1.67 ± 0.14	0.080
BFR/BS (µm^3^/µm^2^/day)	0.48 ± 0.08	0.45 ± 0.08	0.425	0.81 ± 0.18	0.79 ± 0.10	0.668

*Oc.S/BS* osteoclast surface/bone surface, *N.Oc/BS* number of osteoclasts/bone surface, *N.Oc/BV* number of osteoclasts/bone volume, *MS/BS* mineralising surface/bone surface, *sL.Pm* single label perimeter, *dL.Pm* double label perimeter, *MAR* mineral apposition rate, *BFR/BS* bone formation rate/bone surface. The values shown are the mean ± SD values. The p values refer to the difference between genotype groups. Note that for 8-week-old mice data on bone formation parameters were available on 9 *Rin3*^*−*/*−*^ mice

### Biochemical Markers

We found no significant differences between genotype groups in serum concentrations of either PINP or CTX-I values at either 8 weeks or 52 weeks of age. Circulating concentrations of PINP fell significantly with age between 8 weeks and 52 weeks in both genotype groups (both *p* < 0.001). Concentrations of CTX-1 also fell with age and this was significant in the *Rin3*^*−*/*−*^ mice (*p* = 0.03) but not in the wild-type mice (*p* = 0.45) (Table 4).

**Table 4:** Biochemical markers in *Rin3*^*−*/*−*^ and wild-type mice

	8 weeks			52 weeks		
	Wild type (*n* = 10)	*Rin3*^*−*/*−*^ (*n* = 12)	*p* value	Wild type (*n* = 9)	*Rin3*^*−*/*−*^ (*n* = 9)	*p* value
PINP (ng/ml)	8.4 ± 2.2	8.6 ± 2.5	0.584	2.8 ± 1.3	1.5 ± 1.1	0.093
CTX-I (ng/ml)	21.1 ± 5.6	20.1 ± 3.8	0.602	18.5 ± 9.0	13.6 ± 7.4	0.221

The values shown are the mean ± SD values. The p values refer to the difference between genotype groups

### Osteoclast and Osteoblast Cultures

**Fig. 3:**
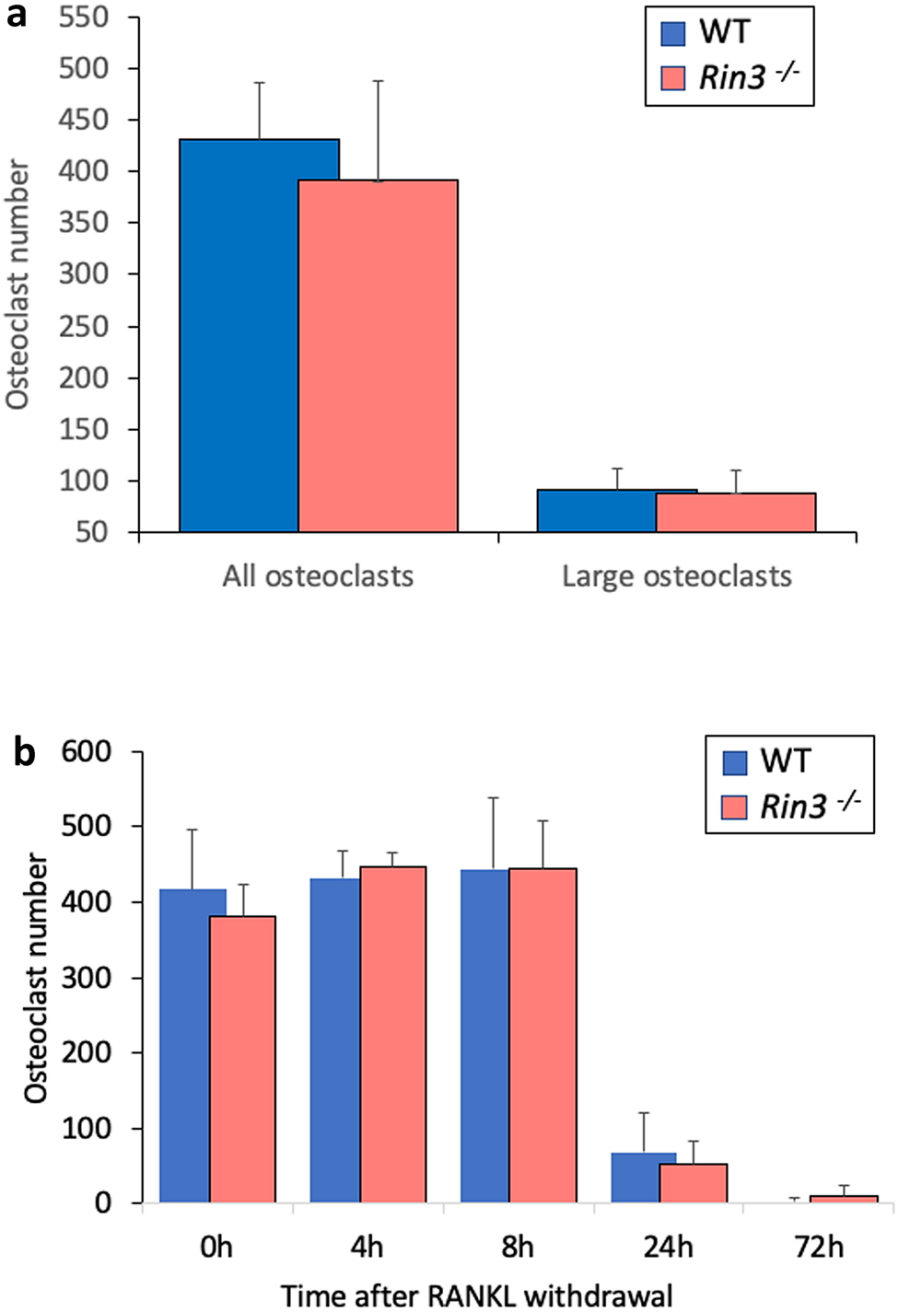
Osteoclast formation and survival in *Rin3*^*−*/*−*^ and wild-type mice. Panel **a**: Osteoclast cultures from wild-type (WT) and *Rin3*^*−*/*−*^ mice, showing total osteoclasts and large osteoclasts (≥ 10 nuclei); Panel **b**: Osteoclast survival following RANKL withdrawal in wild-type (WT) and *Rin3*^*−*/*−*^ mice. There were no significant differences between genotypes, either in osteoclast formation, formation of large osteoclasts or in osteoclast survival at any timepoint. The columns are least square means and bars are standard deviation of the mean for five separate experiments for osteoclast formation and for three separate experiments for osteoclast survival

**Fig. 4:**
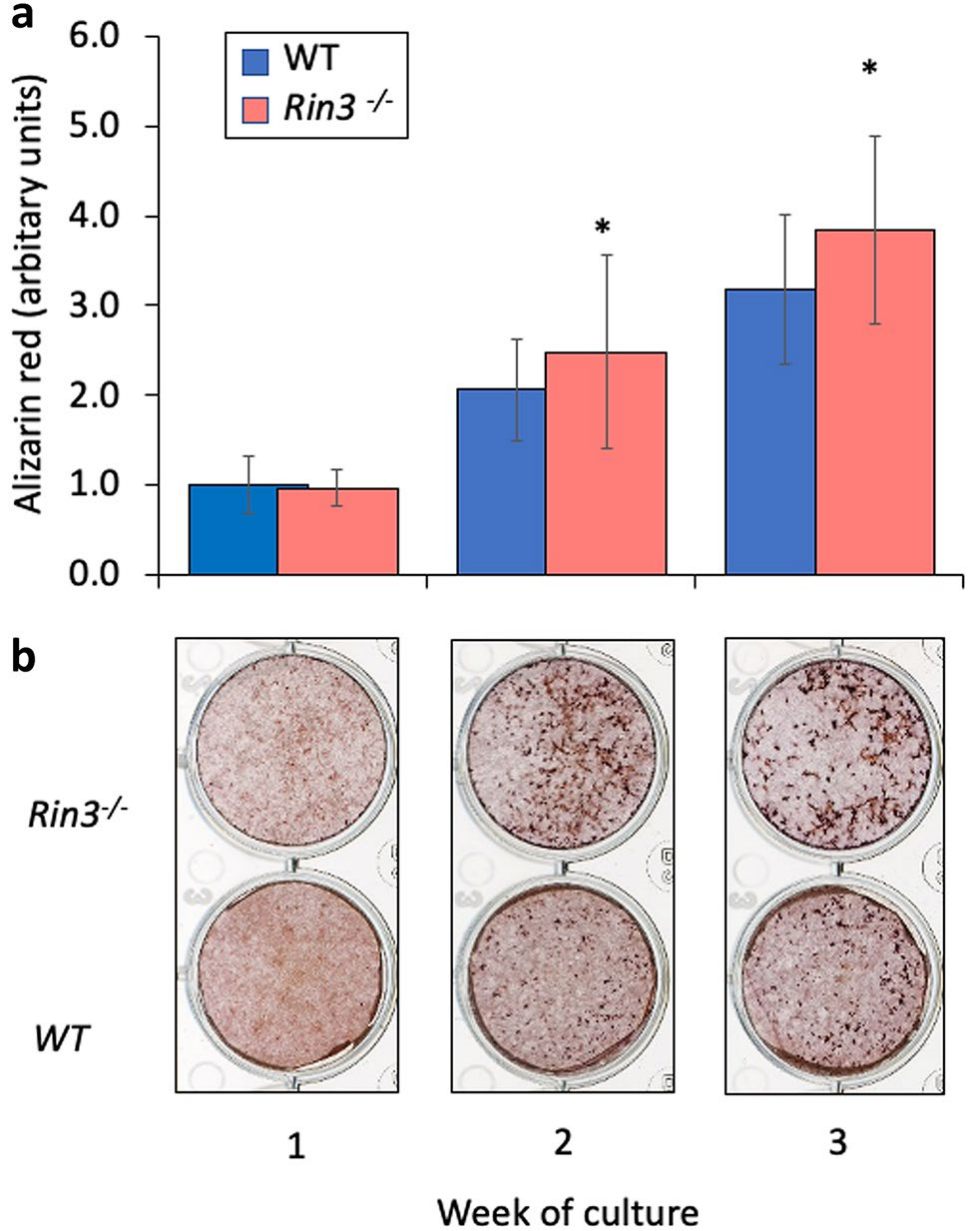
Mineralised nodule formation in *Rin3*^*−*/*−*^ and wild-type mice. Panel **a** Mineralised nodule formation in arbitrary units quantitated by alizarin red staining in cultured calvarial osteoblasts from wild-type (WT) and *Rin3*^*−*/*−*^ mice. The columns are least square means and bars are standard deviations pooled from five separate experiments. *p = 0.02 between genotype groups assessed by GLM ANOVA. Panel **b** representative photomicrographs from bone nodule cultured after 7, 14 and 21 days

We found no differences in M-CSF and RANKL-induced osteoclast formation in cultures prepared from *Rin3*^*−*/*−*^ and wild-type littermates either in terms of total osteoclasts or large osteoclasts with more than ten nuclei (Fig. 3a). There was also no difference between genotype groups in osteoclast survival after withdrawal of RANKL (Fig. 3b). Mineralised nodule formation was slightly but significantly increased in *Rin3*^*−*/*−*^ compared with wild-type mice when data from all experiments were combined (Fig. 4). There was no significant difference in alkaline phosphatase activity in calvarial osteoblasts isolated from *Rin3*^*−*/*−*^ and wild-type mice (data not shown).

### Transcriptome Analysis

Results of the transcriptome analysis assessed by RNA sequencing are summarised in supplementary Fig. S1 and supplementary Table S4. We identified eight transcripts from seven genes that were differentially expressed in calvarial osteoblasts cultured from *Rin3*^*−*/*−*^ and wild-type mice. Only two of the transcripts are known to have a role in bone metabolism and both were upregulated in *Rin3*^*−*/*−*^ mice (*Sfrp2* and *Dlk1*). The *SFPR2* gene has been implicated in syndactyly and brachydactyly in humans [17] but the increased levels of expression seem unlikely to contribute to the phenotype observed in *Rin3*^*−*/*−*^ mice. The *Dlk1* gene is a member of the Notch family which has been implicated as a paracrine and endocrine regulator of bone mass which inhibits osteoblast differentiation [18]. The upregulation of this gene also seems unlikely to contribute to the skeletal phenotype in *Rin3*^*−*/*−*^ mice. We observed no significant differences in patterns of gene expression in osteoclasts generated from bone marrow macrophages between *Rin3*^*−*/*−*^ and wild-type mice when the data were corrected for multiple testing.

## Discussion

The *RIN3* gene was identified as a candidate for susceptibility to PDB by an extended genome-wide association study [2]. The strongest association was with rs10498635 [2] which is in strong linkage disequilibrium with rs117068593, a common missense variant that encodes an arginine to cysteine amino acid substitution at codon 297 (p.R279C) in exon 6 of *RIN3* [8]. Targeted sequencing of *RIN3* subsequently identified thirteen missense variants within the same haplotype block as rs117068593 which, when combined, were strongly over-represented in PDB cases as compared with controls with an odds ratio of greater than 3 [8]. A further study confirmed the association between the common p.R279C variant and PDB in Belgian and Dutch patients and identified six further rare missense variants and PDB [9]. In both studies, *in silico* analysis indicated that p.R279C and many of the rare missense variants were likely to affect protein function, but it remains unclear at present whether they inhibit or enhance the ability of *RIN3* to act as a guanine exchange factor.

The results of the present study are of interest in confirming that *RIN3* plays an important role in regulating bone metabolism, further supporting the candidacy of this gene as a cause of PDB. Trabecular bone volume and trabecular numbers were substantially higher in *Rin3*^*−*/*−*^ mice at 8 weeks of age as compared with wild-type littermates and the difference between genotypes was even greater in mice aged 52 weeks. The increase in penetrance of the phenotype with ageing is of interest in relation to the fact that PDB increases in prevalence with age in humans [19]. It is also consistent with observations in the P394L mouse model of PDB where the phenotype worsened with age and was fully penetrant in 52-week-old mice [20]. Histomorphometric analysis revealed that this was associated with significantly lower osteoclast surfaces at 8 weeks and significantly higher perimeter occupied by single calcein labels at 52 weeks. It is important to emphasise, however, that the differences between genotypes in these parameters were modest and there was no difference between genotype groups in osteoclast numbers expressed as a function of bone surface or bone volume, mineral apposition rate, bone formation rate, or double labelled calcein perimeter at either time point. While analysis of biochemical markers of bone resorption and formation at both 8 weeks and 52 weeks did not reveal a significant difference between groups, the microCT data coupled with the histomorphometry data do indicate that deficiency of *Rin3* causes a relative uncoupling of bone resorption and formation which operates throughout life to cause a markedly increased trabecular bone mass in older mice.

Although trabecular bone mass was markedly higher in *Rin3*^*−*/*−*^ mice, we observed no difference between genotypes in terms of cortical bone parameters except at 52 weeks where the medullary cavity diameter was smaller in *Rin3*^*−*/*−*^ mice compared with wild type, which would be consistent with lower rates of endosteal bone resorption. The contrasting effects of *Rin3* deficiency on trabecular bone phenotype versus cortical bone are unexplained at present, but this finding is not infrequent in mice with targeted inactivation of genes presumably due to site-specific effects of their products on bone metabolism [21–23].

We also studied osteoclast differentiation and osteoclast survival *in vitro* but found no significant differences between genotypes. This is consistent with a model whereby the high bone mass and reduced osteoclast surfaces in *Rin3*^*−*/*−*^ mice were due to a decrease in the amount of bone resorbed per osteoclast rather than by an effect on osteoclast differentiation or survival. The phenotype in *Rin3*^*−*/*−*^ mice is of interest in relation to previous studies by Pavlos and colleagues [24] who conducted phenotyping of mice with targeted inactivation of the small GTPase Rab3D, which is a potential target for *RIN3*. In this study [24], osteoclast differentiation was normal in vitro, but trabecular bone mass was increased in vivo as reported here and this was accompanied by a reduction in eroded surface. In the Pavlos study, it was also of interest that osteoclasts derived from mice with Rab3D deficiency had a defect in resorptive function which was thought to account for the phenotype observed. We did not have the opportunity to study bone resorption by isolated osteoclasts in *Rin3*^*−*/*−*^ mice which is a limitation of the present study, but this work is being planned in the coming months in order to gain greater insight into the role of Rin3 in osteoclast function and bone metabolism. Given that PDB is characterised by increased osteoclast activity and bone resorption, the phenotype we observed in *Rin3*^*−*/*−*^ mice suggests that the missense genetic variants in *RIN3* that are associated with PDB may exert gain-of-function effects on bone resorption although more work will be required to confirm this.

Until now, the role of Rin3 in bone metabolism has been little studied. It is recognised that Rin3 acts as a guanine exchange factor (GEF) for several members of the Rab5 subfamily of GTPases [7, 25, 26]. The Rab proteins play an important role in membrane trafficking in several cell types, and studies by Zhao and colleagues have shown that osteoclasts express many different Rab proteins [10]. There is experimental evidence to show that Rab3D [24] and Rab7 [27] both play a role in osteoclastic bone resorption, but it remains unclear if *Rin3* acts as a GEF for these Rab family members. It is similarly unclear to what extent the Rab5 subfamily members regulate osteoclast function and further work will be required to investigate the targets for *Rin3* in osteoclasts and the mechanisms by which it affects osteoclast function.

The studies reported here are also of interest in relation to the work of Kemp and co-workers who identified *RIN3* as a significant determinant of lower-limb BMD in children [28]. In Kemp’s study, higher BMD values in the lower limbs were associated with the rs754388 variant at genome-wide significant level. The rs754388 variant is in strong linkage disequilibrium with the same direction of effect as the variants that we previously reported to be associated with PDB [8]. At first sight, it seems counter-intuitive that variants associated with a condition characterised by increased osteoclast activity might also be associated with high bone mass in children, but it is possible to speculate that positive coupling between bone resorption and formation during the high turnover state that is characteristic of a growing skeleton could account for this finding. In this regard, variants at the *RIN3* locus do not appear to be associated with BMD as assessed by DEXA in adults [29] or with heel ultrasound properties of bone [30] at a genome-wide significant level.

In conclusion, the present studies confirm that *Rin3* regulates bone mass and bone metabolism in mice with effects on cells of both the osteoclast and the osteoblast lineage, confirming the importance of guanine exchange factors and their targets in regulation of skeletal homeostasis. Our observations also suggest that the variants that predispose to Paget’s disease of bone in humans probably do so by causing a gain-in-function of *RIN3*, but further work will need to be performed to define the molecular mechanisms by which Rin3 affects bone cell activity.

## Supplementary Information

Below is the link to the electronic supplementary material.

(DOCX 2986 kb)
