# Correction to: Targeted Inactivation of *Rin3* Increases Trabecular Bone Mass by Reducing Bone Resorption and Favouring Bone Formation

**DOI:** 10.1007/s00223-021-00849-w

**Published:** 2021-04-19

**Authors:** Mahéva Vallet, Antonia Sophocleous, Anna E. Törnqvist, Asim Azfer, Rob van’t Hof, Omar ME Albagha, Stuart H. Ralston

**Affiliations:** 1 Institute of Genetics and Molecular Medicine, University of Edinburgh, Edinburgh, UK; 2 Department of Life Sciences, School of Sciences, European University Cyprus, Engomi, Cyprus; 3 Centre for Bone and Arthritis Research, Institute of Medicine, University of Gothenburg, Gothenburg, Sweden; 4 Institute of Ageing and Chronic Disease, University of Liverpool, Liverpool, England; 5 College of Health and Life, Hamad Bin Khalifa University, Doha, Qatar

## Correction to: Calcified Tissue International https://doi.org/10.1007/s00223-021-00827-2

The original version of this article unfortunately contained a mistake in Funding section. The correct Funding section is given below.

### Funding

This publication is part of a project that has received funding from the European Research Council (ERC) in the form of an advanced investigator award to SHR under the European Union's Horizon 2020 research and innovation programme (Grant Agreement No. 787270). The publication was also supported in part by a Grant to OMEA from the European Research Council (311723-GENEPAD) and a Grant to SHR from Arthritis Research UK (19799).

