# Supplementary material for: Targeted Inactivation of Rin3 Increases Trabecular Bone Mass by Reducing Bone Resorption and Favouring Bone Formation
Source: Calcif Tissue Int. 2021 Mar 16;109(1):92–102. doi: 10.1007/s00223-021-00827-2 (PMC8225545; doi:10.1007/s00223-021-00827-2)
Supplement: Supplementary file 1 — (DOCX 2986 kb) [file 223_2021_827_MOESM1_ESM.docx]

**Supplementary material**

**Targeted inactivation of *Rin3* increases trabecular bone mass by reducing bone resorption and favouring bone formation**

Mahéva Vallet^1^, Antonia Sophocleous^1,2^, Anna E. Törnqvist^3^, Asim Azfer^1^, Rob van’t Hof^4^, Omar M E Albagha^1^, Stuart H. Ralston^1^

^1.^ Institute of Genetics and Molecular Medicine, University of Edinburgh^; 2.^ Department of Life Sciences, School of Sciences, European University Cyprus; ^3.^ Centre for Bone and Arthritis Research, Institute of Medicine, University of Gothenburg; ^4.^ Institute of Ageing and Chronic Disease, University of Liverpool

**RNA Sequencing methodology and analysis**

*Trimming, processing and mapping of sequencing reads*

The sequencing reads were trimmed for quality at the 3’ end using Cutadapt[1] (version-1.9.dev2). using a quality threshold of 30 and for adapter sequences of the TruSeq stranded RNA Sample Prep Kit (AGATCGGAAGAGC). Reads after trimming were required to have a minimum length of 35. The reads were mapped to the *Mus musculus* genome from Ensembl (assembly GRCm38, annotation version 84) and aligned to the reference genome using STAR [2] (version 2.5.2b) specifying paired-end reads and the option: --outSAMtype BAM Unsorted. All other parameters were left at default.

Reads were assigned to features of type ‘exon’ in the input annotation grouped by gene_id in the reference genome using featureCounts [3] (version 1.5.1) which assigns counts on a ‘fragment’ basis as opposed to individual reads such that a fragment is counted where one or both of its reads are aligned and associated with the specified features. Strandness was set to ‘reverse’ and a minimum alignment quality of 10 was specified.

In addition to the counts matrix used in downstream differential analysis, a matrix of Fragments per Kilobase of transcript per Million mapped reads (FPKM) values was generated, using the rpkm() function of edgeR [4] (version 3.16.5) and normalized effective library sizes. Gene lengths for the FPKM calculation were the number of bases in the exons of each gene (only counting bases once where they occur in multiple exon annotations). Gene names and other fields were derived from input annotation and added to the count/expression matrices.

*Count preprocessing*

The raw counts table was filtered to remove genes consisting predominantly of near-zero counts, filtering on counts per million (CPM) to avoid artefacts due to library depth. Specifically, a row of the expression matrix was required to have values greater than 0.1 in at least 3 samples, corresponding to the smallest sample group as defined by RIN3 genotype status, once any samples were removed (where applicable).

*Analysis of differences between genotype groups*

Differential analysis was carried out with edgeR (version 3.16.5) comparing data from the Rin3^-/-^ and wild type cultures, with correction for the experiment (the animal from which the culture was prepared). Fold changes were estimated as per the default behaviour of edgeR, to avoid artefacts which occur with empirical calculation. Specifically, a small prior count is added to each observation before fitting a model, in proportion to the library size. Log fold-changes are shrunk towards 0, to a greater degree with genes of low count, and infinite fold changes are avoided. Statistical assessment of differential expression was carried out with the quasi-likelihood (QL) F-test.

*Differential gene set analysis*

Differential gene set analysis was carried out with the ROAST method [5] from the Limma package [6] (version 3.30.13) of Bioconductor, using the same models and contrasts as used in differential expression. ROAST was executed using 999,999 rotations (randomisations). Each gene set was annotated with those genes individually differential (in the same direction as indicated for the gene set) to an unadjusted p-value of 0.05.

The following gene sets were used:

1. Gene Ontology Cellular Component, downloaded from Molecular Signatures Database version 5.2 (via Ensembl orthology mappings to mouse, 6/7/2017) [7, 8]
2. MSigDB Canonical pathways, downloaded from Molecular Signatures Database version 5.2 (via Ensembl orthology mappings to mouse, 6/7/2017) [7, 8]
3. KEGG: Kyoto Encyclopedia of Genes and Genomes pathways, downloaded from Molecular Signatures Database version 5.2 (via Ensembl orthology mappings to mouse, 6/7/2017) [9]
4. Gene Ontology Biological Process, downloaded from Molecular Signatures Database version 5.2 (via Ensembl orthology mappings to mouse, 6/7/2017) [7, 8]

Differences in RNA expression between genotype groups were illustrated using volcano plots in which differences in expression are related to the false discovery rate.

**Supplementary Table S1. MicroCT analysis of trabecular bone from the proximal tibial metaphysis in *Rin3^-/-^* and wild type mice.**

|  | **8 weeks** | | | **52 weeks** | | |
| --- | --- | --- | --- | --- | --- | --- |
|  | Wild type  (n=11) | *Rin3^-/-^*  (n=14) | p-value | Wild type  (n=11) | *Rin3^-/-^* (n=10) | p-value |
| BV/TV (%) | 8.5 ± 1.3 | 9.8 ± 1.8 | 0.006 | 6.8 ± 4.7 | 14.0 ± 10.5 | 0.009 |
| Tb.Th (µm) | 37.7 ± 1.7 | 36.9 ± 2.6 | 0.185 | 45.6 ± 5.5 | 49.4 ± 10.4 | 0.163 |
| Tb.Sp (µm) | 231 ± 23 | 211 ± 20 | 0.002 | 342 ± 87 | 261 ± 91 | 0.014 |
| Tb.N (1/mm) | 2.3 ± 0.3 | 2.6 ± 0.3 | <0.001 | 1.5 ± 1.0 | 2.7 ± 1.8 | 0.015 |
| Tb.Pf (1/µm) | 0.033 ± 0.00 | 0.031 ± 0.00 | 0.023 | 0.029 ± 0.01 | 0.020 ± 0.01 | 0.031 |

BV/TV: Bone Volume/Tissue Volume; Tb.Th: Trabecular Thickness; Tb.Sp: Trabecular Separation; Tb.N: Trabecular Number; Tb.Pf: Trabecular Pattern Factor. The values shown are the mean ± sem values. The p-values refer to the difference between genotype groups.

**Supplementary Table S2. MicroCT analysis of trabecular bone from lumbar vertebra 6 in *Rin3^-/-^* and wild type mice.**

|  | **8 weeks** | | | **52 weeks** | | |
| --- | --- | --- | --- | --- | --- | --- |
|  | Wild type (n=11) | *Rin3^-/-^* (n=14) | p-value | Wild type (n=12) | *Rin3^-/-^* (n=11) | p-value |
| BV/TV (%) | 13.6 ± 1.4 | 13.4 ± 2.2 | 0.839 | 21.1 ± 4.1 | 19.4 ± 4.5 | 0.343 |
| Tb.Th (µm) | 34.0 ± 3.1 | 33.3 ± 1.8 | 0.542 | 36.9 ± 3.3 | 38.3 ± 3.4 | 0.328 |
| Tb.Sp (µm) | 204 ± 18 | 205 ± 16 | 0.904 | 159 ± 35 | 165 ± 22 | 0.631 |
| Tb.N (1/mm) | 4.0 ± 0.4 | 4.0 ± 0.5 | 0.975 | 5.7 ± 1.0 | 5.0 ± 0.9 | 0.085 |
| Tb.Pf (1/µm) | 0.018 ± 0.00 | 0.017 ± 0.00 | 0.590 | 0.011 ± 0.01 | 0.012 ± 0.01 | 0.803 |

BV/TV: Bone Volume/Tissue Volume; Tb.Th: Trabecular Thickness; Tb.Sp: Trabecular Separation; Tb.N: Trabecular Number; Tb.Pf: Trabecular Pattern Factor. The values shown are the mean ± sem values. The p-values refer to the difference between genotype groups.

**Supplementary table S3. Detail of RNA sequencing experiment in osteoclasts and osteoblasts**

|  | **Reads** | **Percent of total** |
| --- | --- | --- |
| **Bone marrow derived osteoclasts** | | |
| Total read pairs | 51.4-56.5M | (96.7 - 97.4%) |
| Read pairs after trimming | 49.9-54.9M | (93.4 - 95.4%) |
| Mapped and trimmed read pairs | 47.3-51.4M | (65.8 - 68.8%) |
| Read pairs assigned to features for counting | 32.0-35.1M | (65.8 - 68.8%) |
| Insert size [SD] | 138-150bp | [67-83bp] |
| Mapping quality below threshold | 0-0M | (0 - 0%) |
| Not uniquely mapped | 7.1-7.6M | (14.1 - 15%) |
| Not mapped to feature | 7.7-9.5M | (16.1 - 18.6%) |
| Ambiguous feature mapping | 0.5-0.6M | (1 - 1.2%) |
|  |  |  |
| **Calvarial osteoblasts** | | |
| Total reads pairs | 53.9-58.8M | (96.2 - 96.7%) |
| Read pairs after trimming | 51.9-56.8M | (93.0 - 94.3%) |
| Mapped trimmed read pairs | 48.7-52.9M | (64.8 - 66.8%) |
| Mapped read pairs assigned to features for counting | 31.6-34.5M | (96.2 - 96.7%) |
| Insert size [SD] | 138-150bp | [67-83bp] |
| Mapping quality below threshold | 0-0M | (0 - 0%) |
| Not uniquely mapped | 6.9-8.8M | (13.6 - 17.2%) |
| Not mapped to feature | 8.3-10.3M | (16.1 - 20.1%) |
| Ambiguous feature mapping | 0.4-0.5M | (0.8 - 0.9%) |

Values are the ranges for the median number of reads or the median insert sizes with lowest and highest standard deviation [SD] in the six RNA libraries assessed from each cell type. The range of percentages of total reads from the libraries is also shown. M=million; bp = base pairs

**Supplementary Table S4. Differentially regulated transcripts in calvarial osteoblasts from *Rin3^-/-^* and wild type mice.**

| **Gene** | **Further details** |
| --- | --- |
| *Upregulated transcripts in Rin3^-/-^ osteoblast cultures* | |
| *Gm6394* | Ribosomal protein S11 pseudogene. Function unclear |
| *Gm20746 / Ftl2-ps* | Ferritin-light polypeptide pseudogene 2. Involved in iron transport. Loss of function mutations cause neurodegenerative disease with iron accumulation in the central nervous system [10] |
| *Crispld2* | Cysteine rich secretory protein LCCL domain containing 2. Implicated in craniofacial, kidney and lung development and in host defence against endotoxin [11] |
| *Downregulated transcripts in Rin3^-/-^ osteoblast cultures* | |
| *Figf* | Encodes vascular endothelial growth factor D (VEGF-D). Involved in angiogenesis, lymphangiogenesis and lipid metabolism [12] |
| *Dlk1* | Delta like non-canonical Notch ligand 1. Involved in regulating cell differentiation and tissue repair. Involved in regulating oestrogen deficient bone loss by inhibiting bone formation [13]. |
| *Nrk* | NIK related kinase. A serine/threonine kinase implicated in placental development and mammary gland development through regulation of oestrogen production [14]. |
| *Sfrp2* | Secreted frizzled related protein 2. Involved in multiple developmental processes by inhibiting binding of Wnt to their receptors. Implicated in pathogenesis of syndactyly and brachydactyly [15]. |

**Supplementary figure S1. Differentially expressed genes in RIN3-/- and wild type mice**


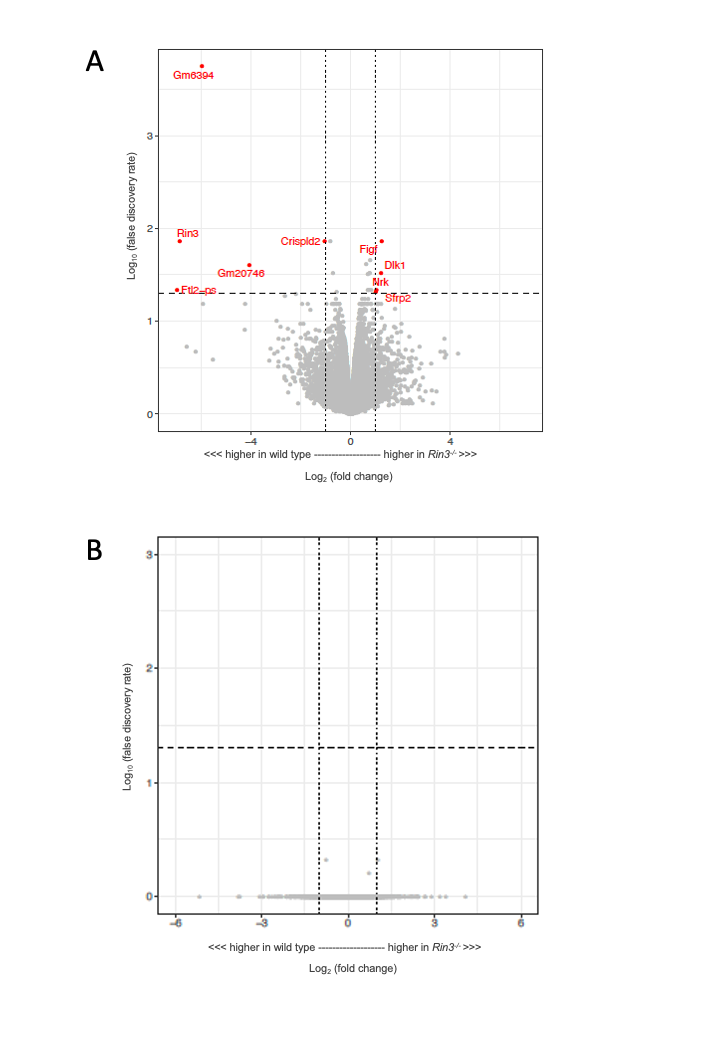


Differentially expressed genes in calvarial osteoblasts are shown in the top panel and those in bone marrow derived osteoclasts in the bottom panel. The volcano plots illustrate log_(2)_ fold change versus -log_(10)_ false discovery rate. The horizontal dashed line represents the specified false discovery rate (FDR) threshold for significance (0.05), dotted lines in both plots represent the specified fold change threshold (2) in both the positive and negative directions. Points passing both thresholds are coloured red, and the top 20 points by FDR are labelled. Note that Gm20746 and Ftl2-ps are likely to be different transcripts from the same gene. No differentially expressed genes were detected in osteoclasts that met the prespecified thresholds although Rin3 expression itself was undetectable in *Rin3^-/-^* cultures (not shown).

**References:**

1. Martin M (2011) Cutadapt Removes Adapter Sequences From High-Throughput Sequencing Reads. EMBnet.journal 17:10-12

2. Dobin A, Davis CA, Schlesinger F, Drenkow J, Zaleski C, Jha S, Batut P, Chaisson M, Gingeras TR (2013) STAR: ultrafast universal RNA-seq aligner. Bioinformatics 29:15-21

3. Liao Y, Smyth GK, Shi W (2014) featureCounts: an efficient general purpose program for assigning sequence reads to genomic features. Bioinformatics 30:923-930

4. Robinson MD, McCarthy DJ, Smyth GK (2010) edgeR: a Bioconductor package for differential expression analysis of digital gene expression data. Bioinformatics 26:139-140

5. Wu D, Lim E, Vaillant F, Asselin-Labat ML, Visvader JE, Smyth GK (2010) ROAST: rotation gene set tests for complex microarray experiments. Bioinformatics 26:2176-2182

6. Ritchie ME, Phipson B, Wu D, Hu Y, Law CW, Shi W, Smyth GK (2015) limma powers differential expression analyses for RNA-sequencing and microarray studies. Nucleic Acids Res 43:e47

7. Ashburner M, Ball CA, Blake JA, Botstein D, Butler H, Cherry JM, Davis AP, Dolinski K, Dwight SS, Eppig JT, Harris MA, Hill DP, Issel-Tarver L, Kasarskis A, Lewis S, Matese JC, Richardson JE, Ringwald M, Rubin GM, Sherlock G (2000) Gene ontology: tool for the unification of biology. The Gene Ontology Consortium. Nat Genet 25:25-29

8. Subramanian A, Tamayo P, Mootha VK, Mukherjee S, Ebert BL, Gillette MA, Paulovich A, Pomeroy SL, Golub TR, Lander ES, Mesirov JP (2005) Gene set enrichment analysis: a knowledge-based approach for interpreting genome-wide expression profiles. Proc Natl Acad Sci U S A 102:15545-15550

9. Ogata H, Goto S, Sato K, Fujibuchi W, Bono H, Kanehisa M (1999) KEGG: Kyoto Encyclopedia of Genes and Genomes. Nucleic Acids Res 27:29-34

10. Chinnery PF, Crompton DE, Birchall D, Jackson MJ, Coulthard A, Lombes A, Quinn N, Wills A, Fletcher N, Mottershead JP, Cooper P, Kellett M, Bates D, Burn J (2007) Clinical features and natural history of neuroferritinopathy caused by the FTL1 460InsA mutation. Brain 130:110-119

11. Wang ZQ, Xing WM, Fan HH, Wang KS, Zhang HK, Wang QW, Qi J, Yang HM, Yang J, Ren YN, Cui SJ, Zhang X, Liu F, Lin DH, Wang WH, Hoffmann MK, Han ZG (2009) The novel lipopolysaccharide-binding protein CRISPLD2 is a critical serum protein to regulate endotoxin function. J Immunol 183:6646-6656

12. Tirronen A, Vuorio T, Kettunen S, Hokkanen K, Ramms B, Niskanen H, Laakso H, Kaikkonen MU, Jauhiainen M, Gordts P, Yla-Herttuala S (2018) Deletion of Lymphangiogenic and Angiogenic Growth Factor VEGF-D Leads to Severe Hyperlipidemia and Delayed Clearance of Chylomicron Remnants. Arterioscler Thromb Vasc Biol 38:2327-2337

13. Figeac F, Andersen DC, Nipper Nielsen CA, Ditzel N, Sheikh SP, Skjodt K, Kassem M, Jensen CH, Abdallah BM (2018) Antibody-based inhibition of circulating DLK1 protects from estrogen deficiency-induced bone loss in mice. Bone 110:312-320

14. Yanagawa T, Denda K, Inatani T, Fukushima T, Tanaka T, Kumaki N, Inagaki Y, Komada M (2016) Deficiency of X-Linked Protein Kinase Nrk during Pregnancy Triggers Breast Tumor in Mice. Am J Pathol 186:2751-2760

15. Morello R, Bertin TK, Schlaubitz S, Shaw CA, Kakuru S, Munivez E, Hermanns P, Chen Y, Zabel B, Lee B (2008) Brachy-syndactyly caused by loss of Sfrp2 function. J Cell Physiol 217:127-137
